# The Institutional Care of the Insane in the United States and Canada
*By Various Writers. Edited by Henry M. Hurd, M.D., LL.D. Illustrated. 4 vols. Demy 8vo. The Johns Hopkins Press, Baltimore, M.D. 1916.


**Published:** 1918-06-01

**Authors:** 


### DR. HENRY HURD'S WONDERFUL BOOK.

### THE INSTITUTIONAL CARE OF THE INSANE IN THE UNITED STATES AND CANADA.*

This monumental work is, in the main, the product of the veteran Dr. Hurd, Emeritus Professor of Psychiatry in the Johns Hopkins University, and formerly Medical Superintendent of the Pontiac State Hospital, who is well known on this side of the Atlantic as the most distinguished of American alienists. Dr. Hurd has retired from active practice, but his abundant energy would not suffer him to be idle, and he has employed his leisure wisely and well in producing this great work, which will be a classic from the day of publication. Devoted as the book is for the most part to the histories of individual American and Canadian institutions, it naturally does not arouse as much interest on this side of the Atlantic as in the land in which it is published. The interest that we can feel in the list of gentlemen who have presided since its erection over the asylum at Kalamazoo must of necessity be languid, but nevertheless the book contains much that is of general, and much that is of permanent interest.

Dr. Hurd traces the history of the gradual recognition of madness, and the gradual introduction in Colonial times of separate treatment for mad people. In the early days of the American colonies, young and struggling as they were, with a sparse population engaged chiefly in agriculture, poverty, bodily infirmity, and mental infirmity were scarcely distinguished from one another, and were treated alike. It was only as the community advanced in population and, as a consequence, in complexity of organisation, that the mad received separate treatment, were separately provided for in legislation, and were separately housed in buildings provided for the purpose. The barbarous treatment of the insane, common to all nations up to within the last three-quarters of a century, lasted longer in America than in any other civilised country, and even as late as the 'eighties of the last century many of the abuses of the old system of restraint and brutality prevailed throughout the United States, long after they had vanished from England, Scotland, and France, thanks to the enlightened labours of Tuke, Pinel, and Conolly.

### Miss Dix a Remarkable Woman.

In America, the introduction and spread of enlightenment in this matter were due to a woman, Miss Dorothea Lynde Dix, who had a very remarkable career, and wielded an influence, and produced results, greater perhaps than any woman not actually seated upon a throne has ever done before or since. Dr. Hurd devotes a long chapter to the history of her labours, and assuredly they are worth describing at length. Born of poor parents in humble circumstances, she had a hard struggle to educate herself, and supported herself in youth by school-teaching. She was hampered throughout life by ill-health, by which her later years were altogether incapacitated, and yet she succeeded in inducing State Legislatures and even Congress to pass legislation at her instance, and in the course of her life was instrumental in founding or enlarging no fewer than thirty State institutions for the insane. Her strength and independence of character showed themselves early. At the age of twelve she left home of her own accord, emancipated herself from subjection to a Harold Skimpole of a father, and placed herself in the household and under the tuition of her grandmother. At nineteen she opened a school, which rapidly became very successful, for girls of well-to-do parents, and soon added to it a charity school for the daughters of the poor. To these labours she added the education of her young brothers and the nursing of the grandmother, with the natural result that her health broke down, and her school had to be abandoned. It was not until she was nearly forty that the death of her grandmother placed her in easy circumstances, and thereafter she devoted herself to a life of philanthropy. By holding a class for the female convicts in a prison she discovered that guilty, innocent, and insane women were mixed up together, and was thus led to investigate the treatment of the insane, to the amelioration of whose lives her own was thenceforth devoted. She began by presenting a petition or memorial to the Legislature of the State of Massachusetts, praying earnestly for the improvement of the treatment of the insane in the State. She was met, of course, by opposition, abuse, ridicule, detraction, and all the weapons by which reformers always are assailed, but she held on, and was completely successful. An Act was passed for the constitution of a State hospital for the insane. After this, she went from State to State, investigating for herself the treatment of the insane, and when she found it defective, as she always did, arousing public opinion to have it ameliorated. At length, in 1848, encouraged by her success with State Legislatures, she attacked Congress itself, and actually induced the Congress of the United States to pass a Bill for the appropriation of ten million acres of the public domain for the care of the indigent insane. The Bill was vetoed by the President, but to have achieved its passage through both Houses of Congress was an astonishing triumph.

### A Foreigner, a Woman and a Dissenter.

Her health being impaired by her exertions, Miss Dix paid a visit to Europe, but when she landed in Scotland, her ruling habit asserted itself. She investigated the treatment of the insane there, and finding it unsatisfactory, flew up to London, inter-

* By Various Writers. Edited by Henry M. Hurd, M.D., LL.D. Illustrated. 4 vols. Demy 8vo. The Johns Hopkins Press, Baltimore, M.D. 1916.

**DR. HURD'S WONDERFUL BOOK**—*(continued)*.

viewed Lord Shaftesbury, the Duke of Argyll, the Home Secretary, and so moved them by her eloquence and earnestness that she procured the appointment of a Royal Commission to inquire into the matter, and the Home Secretary naively deplored, in a speech to the House of Commons, that "the inauguration of so needed a reform should have been left to the initiative of a foreigner, and that foreigner a woman, and that woman a dissenter."

A Practical Pope of 1850.

Having accomplished her purpose in Scotland, she then performed the same feat in the Channel Islands, to which the less reputable keepers of madhouses had been driven from England by legislation. Successful here again, she visited the Continent of Europe, reforming the madhouses wherever she went. She actually induced the Pope to pay a personal visit of inspection to the madhouse in Rome—this, of course, was before his Holiness was the "prisoner of the Vatican"—with the consequence that the administration was immediately reformed. Miss Dix returned to America to find herself regarded as an infallible authority on all matters relating to the care of the insane, and soon after, when the Civil War broke out, she played the part in the hospitals for the wounded that Florence Nightingale played in our own Crimean War. Miss Dix was a wonderful woman. She left a fragrant memory of high-mindedness and disinterestedness, and, what is more, she left the world in many respects very much the better for her labours. She died in 1887, at the age of eighty-five.

Mechanical Restraint in U.S.A.

Dr. Hurd's book is a complete history of the modes of treating the insane in the United States, and deals with every aspect of the subject, far more than can be summarised here. The system of mechanical restraint, which was abolished in this country by the labours and example of Samuel Tuke and Conolly, and in France by those of Pinel, remained in force in America long after it had been discontinued here and in France, and it is amusing now to read the reasons that American alienists gave for declining to follow the English example, with which they could not fail to be familiar. The freeborn American citizen was more violent and destructive when confined in an institution than the tame subject of a monarch, and therefore needed more drastic methods of control. The American insane were more violent and destructive than the English because so many of the insane in America were foreigners who had come from slavish monarchical countries and were not accustomed to liberty: when they landed in a free country they lost their heads. The climate of America was so stimulating that it caused the insanity of those who became insane to assume a peculiarly ferocious type; and so on. The fact is, of course, that the method of mechanical restraint is less trouble, needs less intelligence, gives rise to less anxiety, is much cheaper, and therefore commends itself to the official mind, which abhors trouble, is not very intelligent, and dreads responsibility; and to the mind of the taxpayer, who does not wish the calls on his pocket to be increased. When the effort was once made and the change brought about, it was found that the freeborn but mad American citizen was just as amenable to mild measures as his slavish compeer in monarchical countries, and that the climate of America somehow lost its stimulating character.

The American Medical Treatment.

The history of the medical treatment of the insane forms a very interesting chapter, and shows incidentally how the dominating influence of one strong personality may produce effects that for good or for evil live long after him. Dr. Benjamin Rush was the American Dr. Willis, and taught in America the Willisian doctrine that the first and most necessary aim in the treatment of an insane person was to "catch his eye" and stare him out of countenance, thus obtaining domination over his will. Then the unhappy patient was to be purged, bled, vomited, and blistered until the devil of insanity found his abode so uncomfortable that he took his departure in disgust. It is true that this was not the avowed purpose of the "lowering" treatment of the acutely insane, but it is manifestly the direct descendant and continuation of the doctrine of demoniacal possession. Dr. Hurd passes in review the various fashions—for they are really no more than fashions—that have prevailed in the treatment of the insane, the "lowering" treatment, the "supporting" treatment, the successive fashions of treatment by opium, chloral hydrate, bromide of potash, cannabis indica, paraldehyde, sulphonal, chloralamide, trional, hyoscin, massage, faradism, galvanism, "hospital treatment," which means no more than keeping the patient in bed, and so on and so forth. At length we have come to the conclusion that the malady will run its course, and that our chief effort must be to refrain from harmful meddling. We see that the patient is placed in surroundings most suitable for his case, we give him abundance of food, and we leave Nature to do the rest, pending the time when we shall understand what the morbid process really is, and when we may, perhaps, be able to devise rational means to counteract it.

